# Pan-cancer analysis of whole-genome doubling and its association with patient prognosis

**DOI:** 10.1186/s12885-023-11132-6

**Published:** 2023-07-03

**Authors:** Chie Kikutake, Mikita Suyama

**Affiliations:** Medical Institute of Bioregulation, Kyushu University, Fukuoka, 812-8582 Japan

**Keywords:** Computational biology, Genetics, Genes, Genomics, Mutation, TCGA, PCAWG

## Abstract

**Background:**

Whole-genome doubling (WGD) is a common mutation in cancer. Various studies have suggested that WGD is associated with a poor prognosis in cancer. However, the detailed association between WGD occurrence and prognosis remains unclear. In this study, we aimed to elucidate the mechanism by which WGD affects prognosis using sequencing data from the Pan-Cancer Analysis of Whole Genomes (PCAWG) and The Cancer Genome Atlas.

**Methods:**

Whole-genome sequencing data of 23 cancer types were downloaded from PCAWG project. We defined the WGD event in each sample using the WGD status annotated using PCAWG. We used MutationTimeR to predict the relative timings of mutations and loss of heterozygosity (LOH) in WGD, thus evaluating their association with WGD. We also analyzed the association between WGD-associated factors and patient prognosis.

**Results:**

WGD was associated with several factors, e.g., length of LOH regions. Survival analysis using WGD-associated factors revealed that longer LOH regions and LOH in chr17 were associated with poor prognosis in samples with WGD (WGD samples) and samples without WGD (nWGD samples). In addition to these two factors, nWGD samples showed that the number of mutations in tumor suppressor genes was associated with prognosis. Moreover, we explored the genes associated with prognosis in both samples separately.

**Conclusion:**

The prognosis-related factors in WGD samples differed significantly compared with those in nWGD samples. This study emphasizes the need for different treatment strategies for WGD and nWGD samples.

**Supplementary Information:**

The online version contains supplementary material available at 10.1186/s12885-023-11132-6.

## Background

Cancer, the most common human genetic disease, is caused by the mutations of genes or regulatory regions that control cell growth and division. Mutations in cancer can be classified as single nucleotide variant (SNV), small insertion and deletion (indel), structural variant, copy number variant (CNV), and whole-genome doubling (WGD) [1]. WGD in cancer cells often occurs during mitosis. Inaccurate chromosome segregation during cell division due to checkpoint abnormalities in the G1/S-phase is an important factor for WGD occurrence [2, 3]. Previous studies have shown that 30%–40% of patients with cancer exhibit tumor cells that underwent WGD [4, 5]. The proportion of cells undergoing WGD varies across cancer types. For instance, the proportion is high in lung and ovarian cancers, whereas it is low in glioma and prostate cancers [5]. WGD occurs relatively early in carcinogenesis after the occurrence of oncogenic driver mutation and may promote rapid cancer evolution [5]. The proliferation of tetraploid cells is reportedly suppressed by several signaling pathways, including p53-mediated cell cycle arrest and the Hippo tumor suppressor pathway [6].

WGD is known to confer genetic susceptibility to cancer [7]. Cells with WGD are highly dependent on DNA replication factors and mitotic spindle formation for appropriate division. In WGD cells, the knockout of *KIF18A*, which encodes a mitotic kinesin protein, results in reduced mitotic fidelity and cell death. However, WGD potentially exhibits an advantage for the growth and proliferation of certain tumors [8]. For example, deleterious somatic mutations occurring in the loss of heterozygosity (LOH) regions of diploid cancer cells can lead to cell death; however, WGD might buffer these mutations and chromosomal instability, resulting in cell death evasion. This is exemplified by previous studies showing that WGD carrier samples are associated with a poor prognosis and drug resistance and that the frequency of WGD events is significantly higher in metastatic tumors than in primary tumors in several cancer types [5, 9].

Although the relationship between WGD and prognosis has been described in certain cancer types [5, 9], the mechanism by which samples with WGD contribute to patient survival remains poorly understood. In other words, the conditions under which WGD occurs, relationship between WGD and the cancer evolutionary process, and their impact on patient prognosis remain unclear. The advent of large-scale genome data for various cancer types and samples, which contain information on the occurrence of WGD and mutations before and after the WGD event, enabled the detailed analysis of the mechanism by which WGD affects patient prognosis.

In this study, we analyzed the cancer evolutionary process based on WGD events, with the aim of elucidating how WGD in cancer cells affects patient prognosis. First, we used whole-genome sequencing (WGS), CNV, and ploidy data from the Pan-Cancer Analysis of Whole Genomes (PCAWG) and examined the genomic characteristics associated with WGD events. Next, we analyzed the impact of WGD on patient prognosis and explored genes possibly associated with prognosis. Our results indicate that WGD is associated with longer LOH regions and accumulation of mutations in cancer-related genes within the LOH region. Moreover, we demonstrated that WGD might buffer the impact of cancer-related mutations in the LOH region on poor prognosis. Our study underpins the importance of considering WGD events in cancer diagnosis and treatment.

## Methods

### Dataset

WGS, CNV, purity, ploidy, and clinical data were downloaded from PCAWG project [10]. The samples derived from 23 cancer types were classified into the following 19 cancer types [11]: Biliary (BTCA), Bladder (BLCA), Blood (CLLE, DLBC, and MALY), Bone/Soft tissue (BOCA and SARC), Breast (BRCA), CNS (GBM, LGG, and PBCA), Colon/Rectum (COAD and READ), Esophagus (ESAD), Head and neck (HNSC, ORCA, and THCA), Kidney (KICH, KIRC, KIRP, and RECA), Liver (LICA, LIHC, LINC, and LIRI), Lung (LUAD and LUSC), Myeloid (CMDI and LAML), Ovary (OV), Pancreas (PAEN and PACA), Prostate (EOPC and PRAD), Skin (MELA, SKCM), Stomach (GACA and STAD), and Uterus (CESC and UCEC) (Table 1).

**Table 1:** Classification of 19 cancer types using PCAWG data

Classified cancer types	Cancer types in PCAWG data		Sample size	
			WGD	nWGD
Biliary	BTCA	Biliary tract cancer, Gall bladder cancer/Cholangiocarcinoma	6	6
Bladder	BLCA	Bladder Urothelial cancer/Cholangiocarcinoma	15	8
Blood	CLLE	Chronic Lymphocytic Leukemia	11	191
	DLBC	Lymphoid Neoplasm Diffuse Large B-cell Lymphoma		
	MALY	Malignant Lymphoma		
Bone/Soft tissue	BOCA	Bone Cancer, Ewing Sarcoma	43	55
	SARC	Sarcoma		
Breast	BRCA	Breast Triple Negative Cancer, Breast ER + and HER2-Cancer, Breast Cancer, Lobular Cancer	105	109
CNS	GBM	Brain Glioblastoma Multiforme	39	255
	LGG	Brain Lower Grade Glioma		
	PBCA	Pediatric Brain Cancer		
Colon/Rectum	COAD	Colon Adenocarcinoma	23	37
	READ	Rectum Adenocarcinoma		
Esophagus	ESAD	Esophageal Adenocarcinoma	60	38
Head and neck	HNSC	Head and Neck Squamous Cell Carcinoma	25	80
	ORCA	Oral Cancer		
	THCA	Thyroid papillary carcinoma, Thyroid Cancer, Head and Neck Thyroid Carcinoma		
Kidney	KICH	Kidney Chromophobe	28	161
	KIRC	Kidney Renal Clear Cell Carcinoma		
	KIRP	Kidney Renal Papillary Cell Carcinoma		
	RECA	Renal clear cell carcinoma, Renal Cell Cancer		
Liver	LICA	Liver Cancer	85	261
	LIHC	Liver Hepatocellular carcinoma		
	LINC	Liver Cancer		
	LIRI	Liver Cancer		
Lung	LUAD	Lung Adenocarcinoma	56	30
	LUSC	Lung Squamous cell carcinoma		
Myeloid	CMDI	Chronic Myeloid Disorders	0	50
	LAML	Acute Myeloid Leukemia, Chronic Myelogenous Leukemia		
Ovary	OV	Ovarian Cancer, Ovarian Serous Cystadenocarcinoma	68	45
Pancreas	PACA	Pancreatic Cancer Endocrine Neoplasms, Pancreatic Cancer, Pancreatic Ductal Adenocarcinoma	112	212
	PAEN	Pancreatic Cancer Endocrine Neoplasms, Pancreatic Endocrine Neoplasms		
Prostate	EOPC	Early Onset Prostate Cancer	18	233
	PRAD	Prostate Cancer/Cholangiocarcinoma		
Skin	MELA	Skin Cancer	58	49
	SKCM	Skin Cutaneous Melanoma		
Stomach	GACA	Gastric Cancer	29	46
	STAD	Gastric Adenocarcinoma		
Uterus	CESC	Cervical Squamous Cell Carcinoma	25	46
	UCEC	Uterine Corpus Endometrial Carcinoma		

We also downloaded the whole-exome sequencing (WES), CNV, purity, ploidy, and clinical data of 26 cancer types from The Cancer Genome Atlas (TCGA) data repository [12, 13]. These data were used for validation. Further, we classified these samples into 18 cancer types, similar to the PCAWG data: Bladder (BLCA), Blood (DLBC), Bone/Soft tissue (SARC), Breast (BRCA), CNS (GBM and LGG), Colon/Rectum (COAD and READ), Esophagus (ESCA), Head and neck (HNSC, THCA, and THYM), Kidney (KICH, KIRC, and KIRP), Liver (LIHC), Lung (LUAD and LUSC), Myeloid (LAML), Ovary (OV), Pancreas (PAAD), Prostate (PRAD), Skin (SKCM), Stomach (STAD), and Uterus (CESC and UCEC) (Table 2). No data were available in TCGA for the Biliary cancer type. We used the human reference genome GRCh37 in this study.

**Table 2:** Classification of 18 cancer types using TCGA data

Classified cancer types	Cancer types in PCAWG data		Sample size	
			WGD	nWGD
Bladder	BLCA	Bladder Urothelial cancer/Cholangiocarcinoma	234	151
Blood	DLBC	Lymphoid Neoplasm Diffuse Large B-cell Lymphoma	2	5
Bone/Soft tissue	SARC	Sarcoma	5	4
Breast	BRCA	Breast Triple Negative Cancer, Breast ER + and HER2-Cancer, Breast Cancer, Lobular Cancer	415	528
CNS	GBM	Brain Glioblastoma Multiforme	216	653
	LGG	Brain Lower Grade Glioma		
Colon/Rectum	COAD	Colon Adenocarcinoma	194	258
	READ	Rectum Adenocarcinoma		
Esophagus	ESCA	Esophageal carcinoma	92	68
Head and neck	HNSC	Head and Neck Squamous Cell Carcinoma	20	516
	THCA	Thyroid Papillary Carcinoma, Thyroid Cancer, Head and Neck Thyroid Carcinoma		
	THYM	Thymoma		
Kidney	KICH	Kidney Chromophobe	80	574
	KIRC	Kidney Renal Clear Cell Carcinoma		
	KIRP	Kidney Renal Papillary Cell Carcinoma		
Liver	LIHC	Liver Hepatocellular carcinoma	120	233
Lung	LUAD	Lung Adenocarcinoma	551	419
	LUSC	Lung Squamous Cell Carcinoma		
Myeloid	LAML	Acute Myeloid Leukemia	4	114
Ovary	OV	Ovarian Cancer, Ovarian Serous Cystadenocarcinoma	231	185
Pancreas	PAAD	Pancreatic Adenocarcinoma	40	116
Prostate	PRAD	Prostate Adenocarcinoma	38	427
Skin	SKCM	Skin Cutaneous Melanoma	182	276
Stomach	STAD	Stomach Adenocarcinoma	171	249
Uterus	CESC	Cervical Squamous Cell Carcinoma and Endocervical Adenocarcinoma	202	583
	UCEC	Uterine Corpus Endometrial Carcinoma		

For PCAWG data, we used samples with WGS, CNV, purity, ploidy, and cancer evolution and heterogeneity data that were analyzed via the R package MutationTimeR [1]. For TCGA data, we used samples with WES, CNV, purity, and ploidy data. We analyzed these data in TCGA using MutationTimeR (v.1.00.2) to predict the relative timing of mutations and CNV in WGD. MutationTimeR classifies mutations into the following four groups: early clonal, late clonal, clonal, and subclonal. Early clonal mutations are believed to occur before the WGD event [1]. We defined early and clonal mutations as “early mutations” and late clonal and subclonal mutations as “late mutations.”

We used three types of gene lists: 331 essential genes [14], defined based on their housekeeping function and evolutionary conservation; 723 genes registered in the Cancer Gene Census (CGC) [15]; and 1,217 tumor suppressor genes (TSGs) registered in TSGene [16].

### WGD event in each sample

We defined the WGD event in each sample using the WGD status annotated via PCAWG project and TCGA database. For PCAWG samples, the WGD status was estimated based on the agreement of six types of copy number callers' results [17]. For TCGA samples, the WGD status was estimated using ABSOLUTE algorithm [18] with SNP array and mutational data to generate segmented absolute copy numbers [13].

### WGD event-based LOH definition

We focused on only pre-WGD LOH and regarded it as LOH. We defined a region with a copy number of 0 in either genome as pre-WGD LOH. As the probability that the copy number in either duplicated chromosome becomes 0 via LOH after the WGD event is extremely low, we hypothesized that LOH with a copy number of 0 in either genome occurs before the WGD event and not after the event. For PCAWG data, the LOH, copy-neutral LOH (cn-LOH), and LOH gain regions in CNV data were defined as pre-WGD LOH. For TCGA data, regions with a minor allele copy number of 0 in CNV data annotated via ASCAT [19] were defined as pre-WGD LOH.

### Survival analysis using WGD-associated factors

Cox proportional hazards model in the R survival package (version 3.2–13) was used to estimate hazard ratios (HRs) and their 95% confidence intervals. For PCAWG data, we used cancer type, sex, and age at diagnosis as covariates. For TCGA data, we used cancer stage data, if available, in addition to the abovementioned covariates.

### Statistical analyses

Statistical analyses were performed using R software version 4.0.1 (R Project for Statistical Computing, Vienna, Austria). We used the Wilcoxon rank-sum test to determine significant differences between the two groups and Benjamini–Hochberg (BH) procedure to adjust for multiple testing [20]. Statistical analyses were two-sided, and *P*-values of *P* < 0.05 were considered to indicate statistical significance (*P < 0.05, **P < 0.01, and ***P < 0.001). For prognostic gene exploration, *P*-value < 0.05 and adjusted *P*-values (false discovery rate, FDR) < 0.25 were considered statistically significant in accordance with a previous study [21].

## Results

### Characteristics of pre-WGD event mutations

**Fig. 1:**
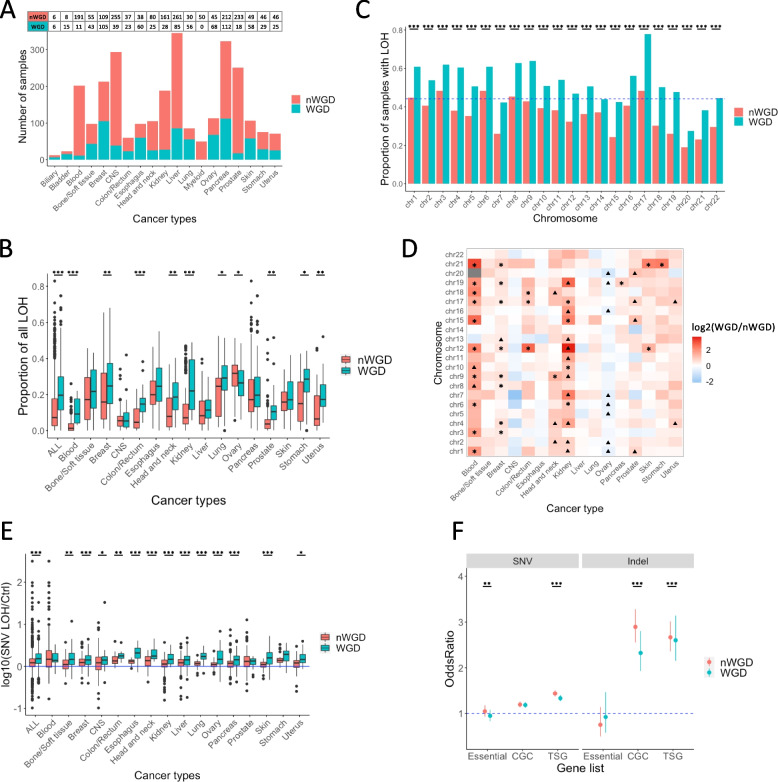
Characteristics of early mutations within the LOH region in samples with and without WGD. **A** Number of samples with and without WGD in the PCAWG data. The horizontal axis represents 19 cancer types. **B** The proportion of total length of LOH region to total genome length for each sample. ALL indicates all samples without distinguishing cancer types. **C** Comparison of the proportion of samples with LOH regions between WGD and nWGD samples. The horizontal axis represents 22 autosomes. **D** The ratio of the proportion of samples with LOH regions in WGD samples to that in nWGD samples (WGD/nWGD). The vertical axis represents 22 autosomes. Black asterisks and triangles indicate *P* < 0.05 and *P* < 0.1, respectively. **E** The ratio of early clonal SNV density within the LOH region to the density outside the LOH region in WGD samples and the ratio of clonal SNV density within the LOH region to the density outside the LOH region in nWGD samples. The vertical axis represents log_10_(SNV density within the LOH region/SNV density outside the LOH region). ALL indicates all samples without distinguishing cancer types. **F** The accumulation of early mutations in genes within the LOH region. The blue dotted line indicates an odds ratio of 1. Odds ratios of > 1 indicate that the mutation is subjected to selective pressure. Red and blue dots represent odds ratios, whereas whiskers represent 95% confidence intervals

To investigate the characteristics of samples with WGD (WGD samples) and samples without WGD (nWGD samples), we calculated the frequency of WGD events in PCAWG samples (Fig. 1A). Of the 2,718 samples derived from 19 cancer types, 806 (29.7%, 806/2,718) samples exhibited WGD. Among these cancer types, Lung displayed the highest proportion of samples with WGD events, (65.1%, 56/86). However, no WGD event could be detected in Myeloid samples. Biliary and Bladder samples exhibited sample sizes of < 30. Therefore, subsequent analyses were performed using all data, excluding Myeloid, Biliary and Bladder sample data.

Previous studies have suggested that WGD can be selected to buffer the deleterious impact of somatic mutations in LOH regions [8]. Therefore, to investigate the deleterious factors associated with WGD occurrence in all cancer types, we determined the proportion of the total length of LOH regions relative to the genome length in each sample. We revealed that the proportion was significantly higher in WGD samples than in nWGD samples when the cancer types were not distinguished (*P* < 2.2e − 16) (Fig. 1B). Further, when the cancer types were distinguished, the proportion of the total length of LOH regions was significantly higher in WGD samples than in nWGD samples for 10 cancer types (Blood, Breast, Colon/Rectum, Head and neck, Kidney, Lung, Ovary, Prostate, Stomach, and Uterus).

Next, we examined the relationship between WGD event and LOH on each chromosome. When cancer types were not distinguished, the proportion of samples with LOH regions was significantly higher in WGD samples than in nWGD samples for all chromosomes (Fig. 1C). In particular, the proportion of samples with LOH on chr17 was the highest among the chromosomes of WGD samples (Fig. 1C). When cancer types were distinguished, the proportion of samples with LOH on chr17 was significantly higher in WGD samples than in nWGD samples (*P* < 0.1) for six cancer types (Blood, Breast, Colon/Rectum, Kidney, Prostate, and Uterus) (Fig. 1D). When we examined the relationship between TSG distribution on each chromosome and the proportion of samples with LOH, we observed, only in the WGD but not in the nWGD samples, that the higher the proportion of samples with LOH on a chromosome, the higher the number of TSGs in the chromosome (*r* = 0.317, *P* = 0.0489) (Supplementary Fig. S1A and B). However, we detected no significant correlations between the proportion of samples with LOH and distribution of essential genes in each chromosome either in WGD or nWGD samples (Supplementary Fig. S1C and D).

Finally, to examine the relationship between WGD event and mutation density in the LOH region, we compared the ratio of mutation density within the LOH region to that outside the LOH region using early clonal mutations, which are believed to occur before the WGD event, in WGD samples and clonal mutations in nWGD samples. When cancer types were not distinguished, we observed that the densities of both SNVs and indels in the LOH region were significantly higher in WGD samples than in nWGD samples (*P* = 2.13e − 53 and *P* = 2.06e − 29, respectively) (Fig. 1E and Supplementary Fig. S2). Moreover, to examine the relationship between WGD event and mutation accumulation in the LOH region, we calculated the odds ratios as follows:

$$Odds\,Ratio = \frac{a/b}{c/d}$$

(a) Total number of mutations in the listed genes (essential genes, genes registered in the CGC, and TSGs registered in TSGene) located in the LOH region, (b) Total number of mutations in all genes, except for the listed genes located in the LOH region, (c) Total number of mutations in the listed genes (essential genes, genes registered in the CGC, and TSGs registered in TSGene) located outside the LOH region, and (d) Total number of mutations in all genes, except for the listed genes located outside the LOH region. For this analysis, we used early clonal mutations in WGD samples and clonal mutations in nWGD samples. The results revealed the selective pressure of mutations within the LOH region in cancer-related genes, but not in essential genes (Fig. 1F). In particular, mutations that disrupt the amino acid sequence (indel, nonsynonymous SNV, and stop-gain SNV) were positively selected in cancer-related genes (Supplementary Fig. S3). These selective pressures were significantly higher in nWGD samples than in WGD samples.

These results indicate that LOH is more likely to occur in chromosomes containing a higher number of TSGs and that the LOH region is longer in WGD samples than in nWGD samples. In addition, WGD samples showed a greater accumulation of mutations in the LOH region than nWGD samples. However, in nWGD samples, mutations are likely to accumulate in TSGs in the LOH region. Subsequent analyses focused on the following four factors: length of LOH region, LOH on chr17, mutation accumulation in TSGs present in the LOH region, and ratio of mutation density within the LOH region to that outside the LOH region.

### Characteristics of post-WGD event mutations

**Fig. 2:**
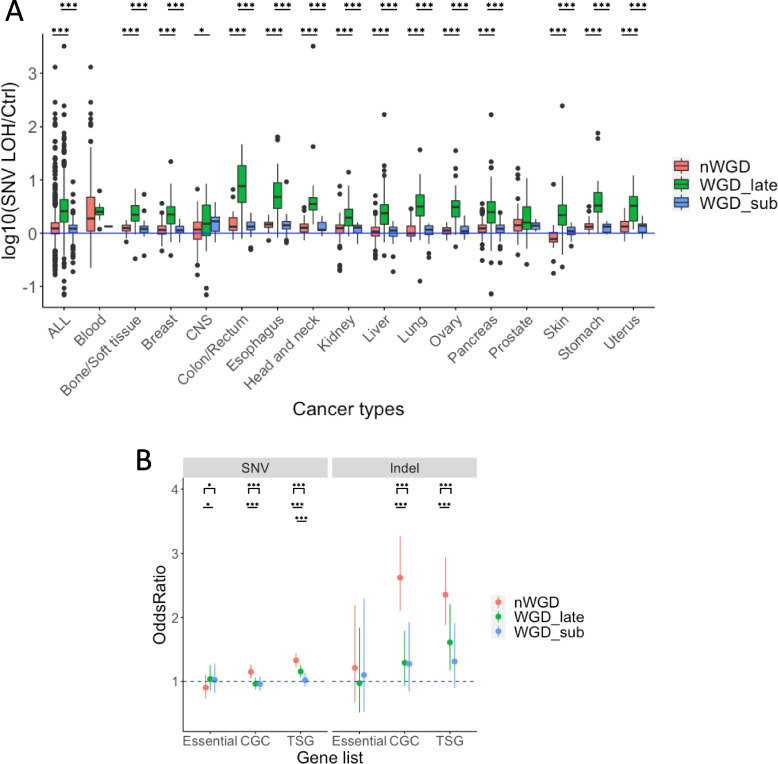
Characteristics of late mutations within the LOH region in samples with and without WGD. **A** The ratio of late clonal and subclonal SNV density within the LOH region to that outside the LOH region in WGD samples and the ratio of subclonal SNV density within the LOH region to that outside the LOH region in nWGD samples. The vertical axis represents log_10_(SNV density within the LOH region/SNV density outside the LOH region). ALL indicates all samples without distinguishing cancer types. **B** The accumulation of late mutations in genes within the LOH region. The blue dotted line indicates an odds ratio of 1. Odds ratios of > 1 indicate that the mutation is subjected to selective pressure. Red and blue dots represent odds ratios, whereas whiskers represent 95% confidence intervals

Herein, we aimed to investigate the mutations in the LOH region after the WGD event. Therefore, we calculated the ratio of mutation density within the LOH region to that outside the LOH region after the WGD event. We used late clonal and subclonal mutations in WGD samples and subclonal mutations in nWGD samples. When cancer types were not distinguished, the ratio of late clonal mutation density was significantly higher than that of subclonal mutation density in WGD samples for both SNVs and indels (*P* = 2.98e − 110 and *P* < 2.2e − 16, respectively) (Fig. 2A and Supplementary Fig. S4). In addition, for both SNVs and indels, the ratio of late clonal mutation density in WGD samples was significantly higher than that of subclonal mutation density in nWGD samples (*P* = 5.06e − 109 and *P* = 1.14e − 111, respectively) (Fig. 2A and Supplementary Fig. S4). When cancer types were distinguished, the same trend was observed for 13 cancer types, except for Blood, CNS, and Prostate (Fig. 2A and Supplementary Fig. S4). These results indicate that mutations are more likely to accumulate in the LOH region immediately after the WGD event.

To examine the accumulation of mutations in the LOH region after the WGD event, we calculated the odds ratios as follows: the number of mutations in each gene group (essential genes, genes registered in the CGC, and TSGs registered in TSGene) against the number of mutations in all genes, except for the listed genes in the LOH regions to those outside the LOH regions in the same way in the previous section “Characteristics of pre-WGD event mutations” (Fig. 2B). For this analysis, we used late clonal and subclonal mutations in WGD samples and subclonal mutations in nWGD samples. We observed that SNVs and indels in genes registered in CGC and TSGene were subjected to stronger selective pressure in nWGD samples than in WGD samples. However, we could not detect any evidence of selective pressure for mutations in the essential genes. Furthermore, we noted a stronger selective pressure for nonsynonymous SNVs and stop-gain mutations than for synonymous mutations in nWGD samples (Supplementary Fig. S5). These results indicate that WGD events reduce the selective pressure for mutations in the LOH region.

Taken together, these results suggest that WGD occurrence buffer deleterious mutations, leading to increased resistance to such mutations. The selective pressure for the amino acid sequence-altering mutations could be reduced as mutations are unlikely to become deleterious in WGD samples. Because mutations in the LOH region could directly affect the phenotype of cancer cells, cells with mutations in TSGs are more likely to remain and continue to grow.

### Relationship between WGD events and patient prognosis

A previous study demonstrated that WGD samples are more significantly associated with a poorer prognosis than nWGD samples [5]. Analysis of WGD event-associated factors in the previous section highlighted that the following four factors may be important for WGD occurrence: length of the LOH region, LOH on chr17, mutation accumulation in TSGs in the LOH region, and ratio of mutation density within the LOH region to that outside the LOH region. Therefore, we hypothesized that these factors are also related to different prognoses of WGD and nWGD samples. To test our hypothesis, we performed survival analysis using these factors as well as the age at diagnosis, sex, and cancer type as covariates. The same analysis was performed using TCGA data to validate these results, except for the ratio of mutation density within the LOH region to that outside the LOH region because TCGA data were derived from WES, not from WGS. Thus, correct mutation density evaluation was not possible.

**Fig. 3:**
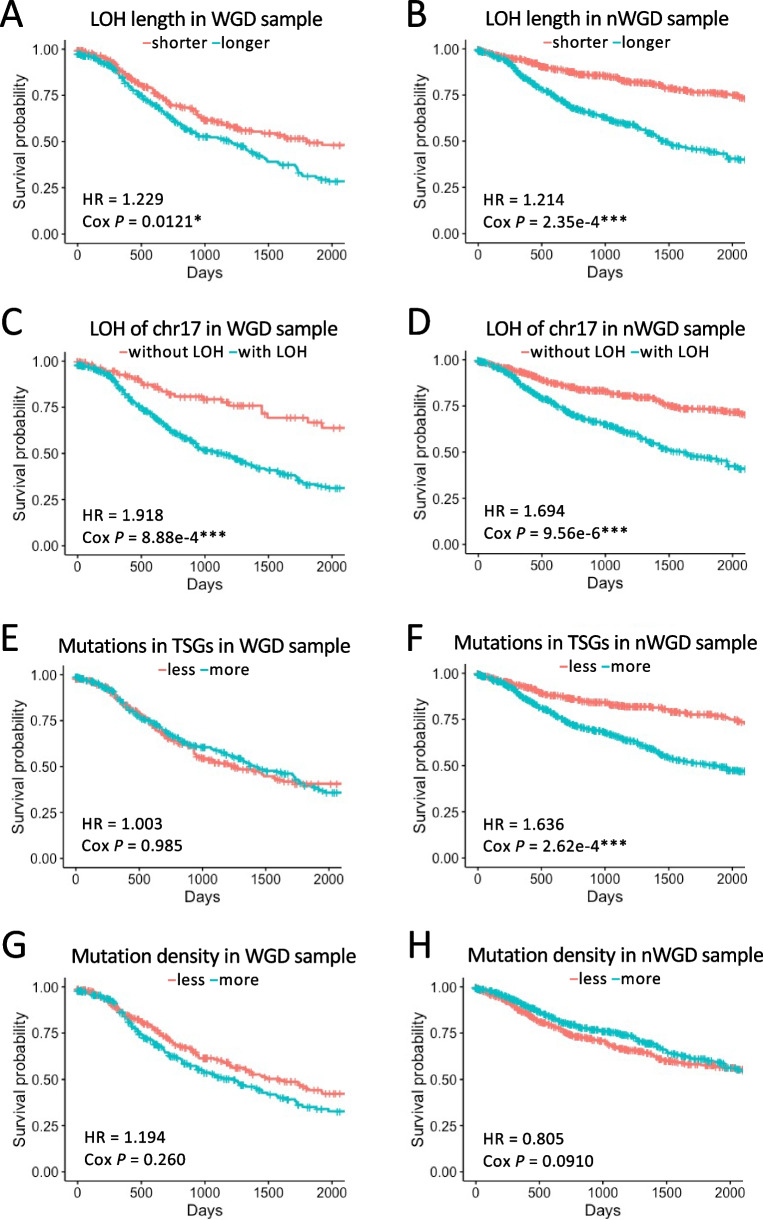
Survival analysis using four WGD occurrence-associated factors. **A** Association between length of LOH region and prognosis in WGD samples. The samples were divided into two groups based on the median total length of LOH region in each sample: longer and shorter groups (blue and red lines, respectively). The horizontal and vertical axes represent survival days (days) and probability, respectively. **B** Association between length of LOH region and prognosis in nWGD samples. The samples were divided into two groups based on the median total length of LOH region in each sample: longer and shorter groups (blue and red lines, respectively). **C** Association between LOH in chr17 and prognosis in WGD samples. The samples were divided into two groups based on the presence or absence of LOH in chr17: groups with and without LOH (blue and red lines, respectively). The horizontal and vertical axes represent survival days (days) and probability, respectively. **D** Association between LOH in chr17 and prognosis in nWGD samples. The samples were divided into two groups based on the presence or absence of LOH in chr17: groups with and without LOH (blue and red lines, respectively). **E** Association between the number of early clonal mutations in TSGs in the LOH region and prognosis in WGD samples. The samples were divided into two groups based on the median number of mutations in TSGs in the LOH region of each sample: groups with more and less mutations (blue and red lines, respectively). **F** Association between the number of clonal mutations in TSGs in the LOH region and prognosis in nWGD samples. The samples were divided into two groups based on the median number of mutations in TSGs in the LOH region of each sample: groups with more and less mutations (blue and red lines, respectively). **G** Association between the ratio of early clonal mutation density within the LOH region to that outside the LOH region and prognosis in WGD samples. The samples were divided into two groups based on the median ratio in each sample: higher and lower ratio groups (blue and red lines, respectively). **H** Association between the ratio of clonal mutation density within the LOH region to that outside the LOH region and prognosis in nWGD samples. The samples were divided into two groups based on the median ratio in each sample: higher and lower ratio groups (blue and red lines, respectively)

Our results demonstrated that both WGD and nWGD samples with longer LOH regions and LOH on chr17 were significantly associated with a poor prognosis (Fig. 3A–D). However, TCGA data failed to confirm the significant association between the LOH on chr17 and prognosis in WGD samples (Supplementary Fig. S6A–D). In WGD samples, no significant association was observed between the number of mutations in TSGs in the LOH region and prognosis (Fig. 3E and Supplementary Fig. S6E). In contrast, in nWGD samples, the higher mutation number in the TSGs in the LOH region was significantly associated with poor prognosis (Fig. 3F and Supplementary Fig. S6F). No difference was noted in the prognosis of the two groups based on the ratio of mutation density within the LOH region to that outside the LOH region (Fig. 3G and H).

In addition, based on late clonal and subclonal mutations in the LOH region, we performed survival analysis using two factors (the odds ratio of mutation in TSGs in the LOH region and the ratio of mutation density within the LOH region to that outside the LOH region). However, these factors were not significantly associated with prognosis (Supplementary Figs. S7A–F, and S8A–C).

In summary, these results indicate that the length of the LOH region can affect patient prognosis both in WGD and nWGD samples. In addition, the number of mutations in TSGs in the LOH region was an important factor for prognosis in nWGD samples.

### Prognostic gene exploration in samples with and without WGD

To explore the prognostic genes in samples with and without WGD, we performed survival analysis using two factors: length of LOH region and TSG mutations in the LOH region. Although all previous analyses were primarily conducted using PCAWG data, we relied on TCGA data for the current analysis due to the availability of relatively sufficient number of samples for each cancer type. We also used PCAWG data to validate the results of this analysis.

**Fig. 4:**
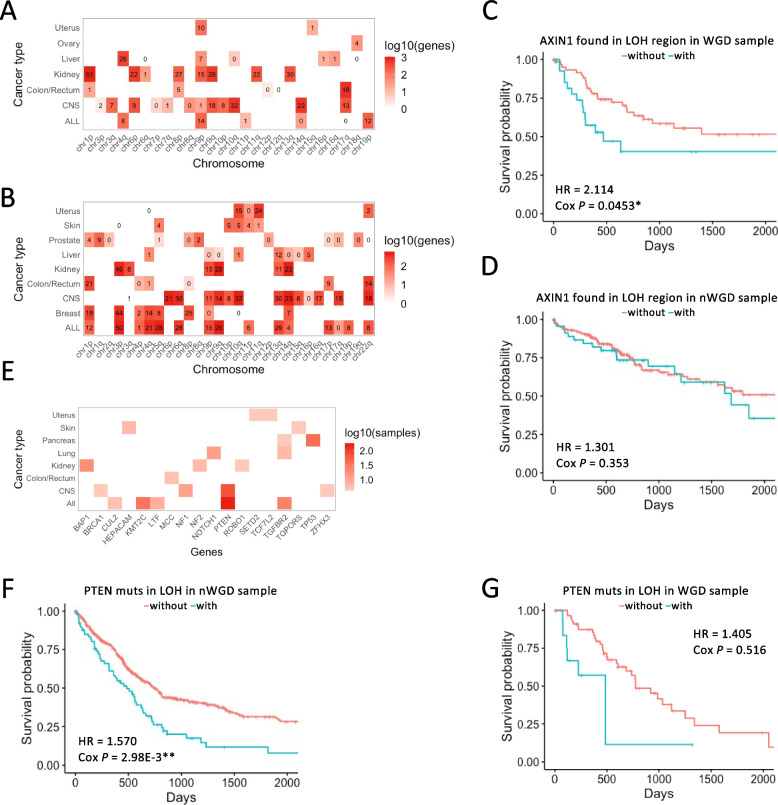
Prognostic gene exploration using TCGA data. **A** The number of prognosis-related genes in the LOH region in WGD samples based on TCGA data. These genes showed a worse prognosis when found in the LOH region than when not found in the LOH region. The red square indicates the number of genes. Numbers in the squares represent the number of TSGs. ALL indicates all samples without distinguishing cancer types. **B** The number of prognosis-related genes located in the LOH region in nWGD samples based on TCGA data. ALL indicates all samples without distinguishing cancer types. **C** Association between *AXIN1* in the LOH region and prognosis in WGD samples based on Liver cancer in TCGA data. The samples were divided into two groups based on the presence or absence of *AXIN1* in the LOH region: groups with and without *AXIN1* in the LOH region (blue and red lines, respectively). The horizontal axis represents survival days (days), whereas the vertical axis represents survival probability. **D** Association between *AXIN1* in the LOH region and prognosis in nWGD samples based on Liver cancer in TCGA data. The samples were divided into two groups based on the presence or absence of *AXIN1* in the LOH region: groups with and without *AXIN1* in the LOH region (blue and red lines, respectively). **E** The number of samples with mutations in prognosis-related TSGs detected in the LOH region of WGD samples based on TCGA data. Samples with mutations in these genes located in the LOH region exhibit a worse prognosis than other samples. The red square indicates the number of samples with mutations in genes located in the LOH region. ALL indicates all samples without distinguishing cancer types. **F** Association between *PTEN* mutations in the LOH region and prognosis in nWGD samples based on CNS samples in TCGA data. The samples were divided into two groups based on the presence or absence of *PTEN* mutations detected in the LOH region: groups with and without *PTEN* mutations (blue and red lines, respectively). **G** Association between *PTEN* mutations in the LOH region and prognosis in WGD samples based on CNS samples in TCGA data. The samples were divided into two groups based on the presence or absence of *PTEN* mutations detected in the LOH region: groups with and without *PTEN* mutations (blue and red lines, respectively)

First, we examined the association between genes frequently located in LOH regions and prognosis based on the length of LOH region. For each cancer type in WGD and nWGD samples, we established two groups based on the percentiles of LOH length: ≥ 75^th^ and ≤ 25^th^ percentile groups (H and L, respectively). For each sample, we extracted genes from the LOH region. We then counted the number of samples with genes in the LOH region between every two groups (*n*_H_ and *n*_L_), normalized the sample counts of the genes between every two groups (*n'*_H_ and *n'*_L_), calculated their differences (∆[*n'*_H_ − *n'*_L_]), and extracted genes with the top 25% of the differences. Survival analysis was performed using the presence or absence of the extracted genes in the LOH regions as a variable. We extracted genes with a significant difference in prognosis (FDR < 0.25, *P* < 0.05, HR > 1) and examined the extracted gene distribution in each chromosome arm (Fig. 4A and B, Supplementary Fig. S9A, and B). In WGD samples, 35 chromosome arms for 6 cancer types contained at least 1 extracted gene, whereas in nWGD samples, 64 chromosome arms for 8 cancer types contained at least 1 extracted gene. The results showed that the LOH regions in the WGD samples with a significant impact on patient prognosis did not always correspond to those in nWGD samples. For example, in WGD samples, the LOH of the cancer suppressor gene *AXIN1* in chromosome 16p13.3 of liver cancer cells could lead to a poor prognosis, whereas it did not affect prognosis in nWGD samples (Fig. 4C and D). The relationship between the LOH of *AXIN1* in chr16 and prognosis was also confirmed by Liver samples in the PCAWG data (Supplementary Fig. S10). Within 16p13.3, the LOH of 16 genes (*POLR3K*, *SNRNP25*, *RHBDF1*, *MPG*, *NPRL3*, *HBZ*, *HBM*, *HBA2*, *HBA1*, *HBQ1*, *LUC7L*, *FAM234A*, *RGS11*, *ARHGDIG*, *PDIA2*, and *AXIN1*) (Supplementary Fig. S11) was associated with patient prognosis in Liver samples.

Next, we focused on TSG mutations in the LOH region of nWGD samples. To examine the association between the TSG mutations in the LOH region and patient prognosis, we performed survival analysis using the presence or absence of TSG mutations in LOH regions as a variable (Fig. 4E and Supplementary Fig. S12). Among 1,217 TSGs, 853 genes had mutations in the LOH region of at least 1 sample. The most frequent mutation was identified in *TP53*, located in the LOH region of 878 samples. Mutations in 16 TSGs of 7 cancer types correlated with a poor prognosis. For example, in CNS samples, mutations in *PTEN* were significantly associated with a poorer prognosis in nWGD samples, but not in WGD samples (Fig. 4F and G). The relationship between mutations in *PTEN* and prognosis was also confirmed by CNS samples in the PCAWG data (Supplementary Fig. S13).

In summary, the distinctive features of WGD and nWGD samples, such as LOH regions, genes in LOH regions, and mutations, could be prognostic factors for patients with cancer.

## Discussion

WGD is a relatively frequent event in cancer cells and is pivotal for cancer evolution [7]. In this study, based on the WGD event, we estimated the evolutionary process in cancer and explored prognostic factors. First, we analyzed the association between WGD or nWGD samples and four factors, namely length of LOH region, LOH in chr17, mutation accumulation in TSGs in the LOH region, and ratio of mutation density within the LOH region to that outside the LOH region. In the LOH region of nWGD samples, both clonal and subclonal mutations were significantly accumulated in cancer-related genes. This might be because cancer-related mutations with a single copy of DNA are likely to have an advantage for cell growth. In contrast, our results demonstrated that the selective pressure for mutations in cancer-related genes in the LOH region of WGD samples reduced after the WGD event. This is potentially because various mutations are prone to occur within the duplicated LOH region, and mutations in the duplicated genome may not provide an advantage for the growth and proliferation of cancer cells.

**Fig. 5:**
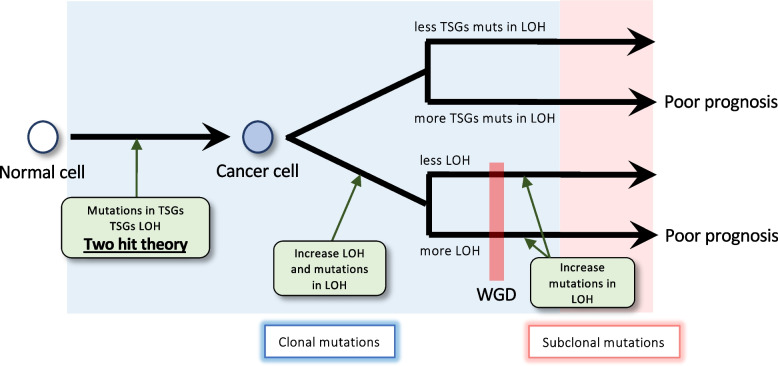
WGD event-based cancer evolution model. The predicted evolutionary process from normal to cancer cells and its relationship with prognosis. The red vertical line represents the timing of the occurrence of WGD event. Blue highlights indicate the timing of the occurrence of clonal mutations, whereas red highlights indicate the timing of the occurrence of subclonal mutations

Based on these results, we proposed a cancer evolution model in samples with and without WGD events (Fig. 5). WGD in cancer cells is likely to occur due to an increase in the length of the LOH region and number of mutations in this region. In WGD samples, longer LOH regions could be associated with a poorer prognosis. After WGD, as the abovementioned deleterious effects are likely buffered by the duplicated genome, the number of mutations in the LOH region increases. In contrast, in nWGD samples, the number of TSG mutations increases in the LOH region, which could be associated with a poorer prognosis. In part, this is because TSG mutations within the LOH region are likely favorable for cancer growth in nWGD samples.

A previous study showed that WGD events in cancer cells result in increased resistance to deleterious mutations, copy number alterations, and chromosomal instability (CIN) [22]. These findings were supported by our results showing that mutations accumulated in the LOH region after WGD events were not associated with prognosis. In this study, survival analysis revealed that longer LOH regions were associated with poor prognosis both in WGD and nWGD samples, potentially due to its association with CIN itself [23–25]. Unlike nWGD samples, WGD samples indicated that TSG mutations in the LOH region before WGD had no impact on prognosis. In a previous study about the conditions of the occurrence of WGD event, it was reported that WGD is more likely to occur when a relatively higher number of mutations occur [8]. Therefore, no difference in prognosis was considered according to TSG mutations because WGD buffered the effects of deleterious mutations. Based on the current and previous studies describing that WGD was associated with poor prognosis [5], it is important to obstruct the WGD event in cancer cells for developing an effective therapeutic strategy. In addition, different therapeutic strategies would be required for WGD samples, which exhibit acquired resistance to various genomic alterations, and nWGD samples, which are susceptible to the effects of TSG mutations in the LOH region.

During the exploration of prognostic factors based on the length of the LOH region, several genes in this region were extracted as prognosis-related genes in both WGD and nWGD samples. Of these genes, survival analysis using both TCGA and PCAWG data revealed that 16 genes in the LOH region at 16p13.3 in Liver cancer cells were significantly associated with a poor prognosis only in WGD samples. Previous studies have reported LOH at 16p13.3 in papillary neoplasms of breast [26] and thyroid [27] cancers. These genomic alterations of the 16p13.3 locus were associated with a poor prognosis [26, 27]. *AXIN1*, a known TSG, was among the 16 genes located at 16p13.3. AXIN1 is involved in the Wnt/β-catenin signaling pathway, and loss-of-function mutations in *AXIN1* in liver cancer are associated with an enrichment of signals related to the cell cycle as well as a more aggressive phenotype [28]. Previous studies have shown that cn-LOH as well as deletion could be associated with poor prognosis in cancer [29, 30]. This is consistent with the finding that *AXIN1* at 16p13.3 is located in cn-LOH region in WGD samples.

Furthermore, *PTEN* mutations in the LOH region of the CNS were associated with a poor prognosis only in nWGD samples. *PTEN* is a TSG, a negative regulator of the PI3K/Akt/mTOR signaling cascade, and is central to controlling various cellular functions, such as proliferation, survival, and metabolism of cells [31, 32]. Previous studies have shown that *PTEN* is frequently mutated in the LOH region in various cancer types [33]. Our finding indicating differences in *PTEN* mutations that affect patient prognosis between WGD and nWGD samples is an example of buffering the effects of deleterious mutations in the LOH region by WGD [8]. It is essential to consider the WGD event to analyze the influence of mutations on cancer pathogenesis as mutations with different copy numbers might affect cancer pathogenesis differently.

Intratumor heterogeneity is a hallmark of cancer, representing that a high number of clones harbor various mutations within a single tumor. Therefore, WGD may occur in partial cells but not in all tumor cells. In this study, we used PCAWG and TCGA data regarding WGD occurrence in each sample, without considering the proportion of cells with WGD. To evaluate the WGD-based cancer evolution more accurately, cancer cells should be analyzed individually; e.g., using single-cell analysis or multiregional sampling analysis. Tracing genomic alterations in individual cells over the entire cancer evolution might reveal novel prognosis-related events that could not be identified in this study.

In addition to the WGD event discussed in this analysis, the cancer evolutionary process has been analyzed through various approaches, such as epigenetic modification [34], chromatin structure alteration [35], and extrachromosomal DNA [36]. Although these individual findings reportedly reveal important factors affecting cancer evolution, such factors are intricately intertwined in cancer cells. Therefore, if comprehensive data of cancer genomes are available with a sufficient sample size in the future, we could estimate the evolutionary process based on the integration of various factors and determine detailed characteristics related to the evolutionary process. Such analysis would lead to the discovery of novel key players in the evolutionary process of cancer and patient prognosis.

## Conclusions

Herein, we provided new insights into the relationship between WGD-based cancer evolution and patient prognosis. To date, only a few studies have focused on this relationship. We revealed that it is possible to identify new prognostic factors by considering WGD in patients with cancer. Finally, this study emphasizes the need for careful consideration of WGD events in cancer diagnosis and treatment.

## Supplementary Information

**Additional file 1:** **Supplementary Figure S1.** The proportion of samples with LOH in each chromosome and TSG and essential gene distribution. **Supplementary Figure S2.** The ratio of early clonal indel density within the LOH region to that outside the LOH region in WGD samples and the ratio of clonal indel density within the LOH region to that outside the LOH region in nWGD samples. **Supplementary Figure S3.** Early mutation accumulation in genes within the LOH region. **Supplementary Figure S4.** The ratio of late clonal and subclonal indel density within the LOH region to that outside the LOH region in WGD samples and the ratio of subclonal indel density within the LOH region to that outside the LOH region in nWGD samples. **Supplementary Figure S5.** Late mutation accumulation in genes within the LOH region. **Supplementary Figure S6.** Survival analysis using WGD occurrence-associated factors in case of early mutations based on TCGA data. **Supplementary Figure S7.** Survival analysis using WGD occurrence-associated factors in case of late mutations. **Supplementary Figure S8.** Survival analysis using WGD occurrence-associated factors in case of late mutations based on TCGA data. **Supplementary Figure S9.** Prognostic gene exploration using PCAWG data. **Supplementary Figure S10.** Association between AXIN1 in the LOH region and prognosis in WGD samples using PCAWG data. **Supplementary Figure S11.** Extracted prognosis-related genes in the 16p.13.3 locus. **Supplementary Figure S12.** Number of samples with prognosis-related TSGs in the LOH region of WGD samples using PCAWG data. **Supplementary Figure S13.** Association between PTEN mutations in LOH and prognosis in nWGD samples using PCAWG data.

## Data Availability

All datasets are freely available from public databases. The study results are mainly based on data obtained from PCAWG (https://dcc.icgc.org/pcawg/) and TCGA (https://portal.gdc.cancer.gov/). We used UCSC genome browser (http://genome.ucsc.edu/). Further information and requests for datasets and scripts generated in the present study should be directed to and will be fulfilled by the Lead Contact, Mikita Suyama (mikita@bioreg.kyushu-u.ac.jp).

## Abbreviations

BLCA: Bladder urothelial cancer/cholangiocarcinoma
BOCA: Bone cancer, ewing sarcoma
BRCA: Breast triple negative cancer, breast ER + and HER2-cancer, breast cancer, lobular cancer
BTCA: Biliary tract cancer, gall bladder cancer/cholangiocarcinoma
CESC: Cervical squamous cell carcinoma
CGC: Cancer Gene Census
CIN: Chromosomal instability
CLLE: Chronic lymphocytic leukemia
CMDI: Chronic myeloid disorders
CNV: Copy number variant
COAD: Colon adenocarcinoma
DLBC: Lymphoid neoplasm diffuse large B-cell lymphoma
EOPC: Early onset prostate cancer
ESAD: Esophageal adenocarcinoma
GACA: Gastric cancer
GBM: Brain glioblastoma multiforme
HNSC: Head and neck squamous cell carcinoma
HR: Hazard ratio
KICH: Kidney chromophobe
KIRC: Kidney renal clear cell carcinoma
KIRP: Kidney renal papillary cell carcinoma
LAML: Acute myeloid leukemia, chronic myelogenous leukemia
LGG: Brain lower grade glioma
LICA: Liver cancer
LIHC: Liver hepatocellular carcinoma
LINC: Liver cancer
LIRI: Liver cancer
LOH: Loss of heterozygosity
LUAD: Lung adenocarcinoma
LUSC: Lung squamous cell carcinoma
MALY: Malignant lymphoma
MELA: Skin cancer
ORCA: Oral cancer
OV: Ovarian cancer, ovarian serous cystadenocarcinoma
PACA: Pancreatic cancer endocrine neoplasms, pancreatic cancer, pancreatic ductal adenocarcinoma
PAEN: Pancreatic cancer endocrine neoplasms, pancreatic endocrine neoplasms
PBCA: Pediatric brain cancer
PCAWG: Pan-Cancer Analysis of Whole Genomes
PRAD: Prostate cancer/cholangiocarcinoma
READ: Rectum adenocarcinoma
RECA: Renal clear cell carcinoma, renal cell cancer
SARC: Sarcoma
SKCM: Skin cutaneous melanoma
SNV: Single nucleotide variant
STAD: Gastric adenocarcinoma
TCGA: The Cancer Genome Atlas
THCA: Thyroid papillary carcinoma, thyroid cancer, head and neck thyroid carcinoma
TSG: Tumor suppressor gene
UCEC: Uterine corpus endometrial carcinoma
WES: Whole-exome sequencing
WGD: Whole-genome doubling
