# Supplementary material for: Pan-cancer analysis of whole-genome doubling and its association with patient prognosis
Source: BMC Cancer. 2023 Jul 3;23:619. doi: 10.1186/s12885-023-11132-6 (PMC10318685; doi:10.1186/s12885-023-11132-6)
Supplement: Supplementary file 1 — Additional file 1: Supplementary Figure S1. The proportion of samples with LOH in each chromosome and TSG and essential gene distribution. Supplementary Figure S2. The ratio of early clonal indel density within the LOH region to that outside the LOH region in WGD samples and the ratio of clonal indel density within the LOH region to that outside the LOH region in nWGD samples. Supplementary Figure S3. Early mutation accumulation in genes within the LOH region. Supplementary Figure S4. The ratio of late clonal and subclonal indel density within the LOH region to that outside the LOH region in WGD samples and the ratio of subclonal indel density within the LOH region to that outside the LOH region in nWGD samples. Supplementary Figure S5. Late mutation accumulation in genes within the LOH region. Supplementary Figure S6. Survival analysis using WGD occurrence-associated factors in case of early mutations based on TCGA data. Supplementary Figure S7. Survival analysis using WGD occurrence-associated factors in case of late mutations. Supplementary Figure S8. Survival analysis using WGD occurrence-associated factors in case of late mutations based on TCGA data. Supplementary Figure S9. Prognostic gene exploration using PCAWG data. Supplementary Figure S10. Association between AXIN1 in the LOH region and prognosis in WGD samples using PCAWG data. Supplementary Figure S11. Extracted prognosis-related genes in the 16p.13.3 locus. Supplementary Figure S12. Number of samples with prognosis-related TSGs in the LOH region of WGD samples using PCAWG data. Supplementary Figure S13. Association between PTEN mutations in LOH and prognosis in nWGD samples using PCAWG data. [file 12885_2023_11132_MOESM1_ESM.pdf]

Fig. S1
C Kikutake et. al

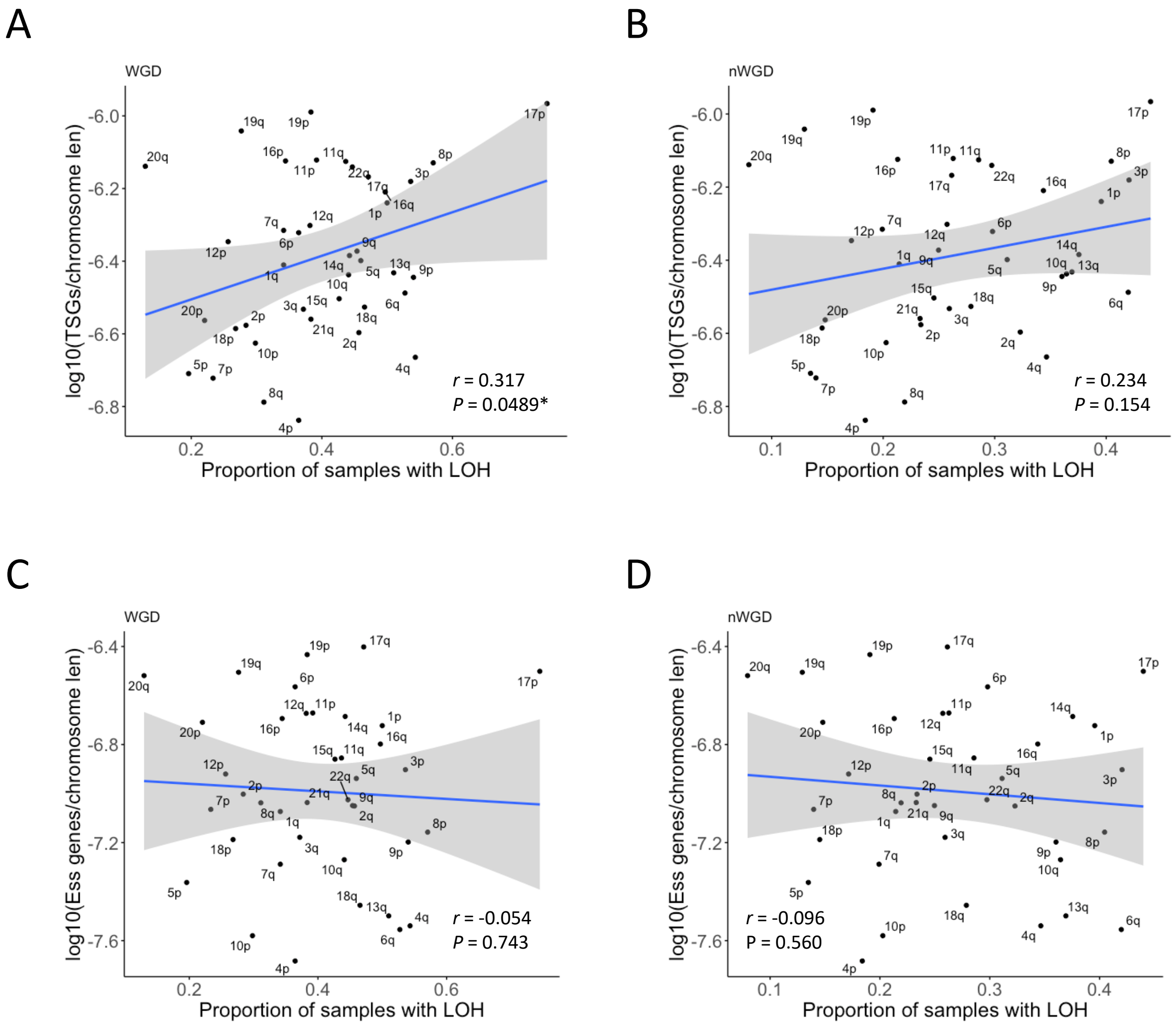

Supplementary Figure S1. The proportion of samples with LOH in each chromosome and TSG and essential gene distribution.

(A) The proportion of samples with LOH in each chromosome and log10 value (total number of TSGs/length of chromosome region) in WGD samples. The chromosomes were divided into long and short arms. Correlation coefficients (r) are indicated using P-values, representing the results of testing for zero correlation. (B) The proportion of samples with LOH in each chromosome and log10 value (total number of TSGs/length of chromosome region) in nWGD samples. (C) The proportion of samples with LOH in each chromosome and log10 value (total number of essential genes/length of chromosome region) in WGD samples. (D) The proportion of samples with LOH in each chromosome and log10 value (total number of essential genes/length of chromosome region) in nWGD samples.

Fig. S2
C Kikutake et. al

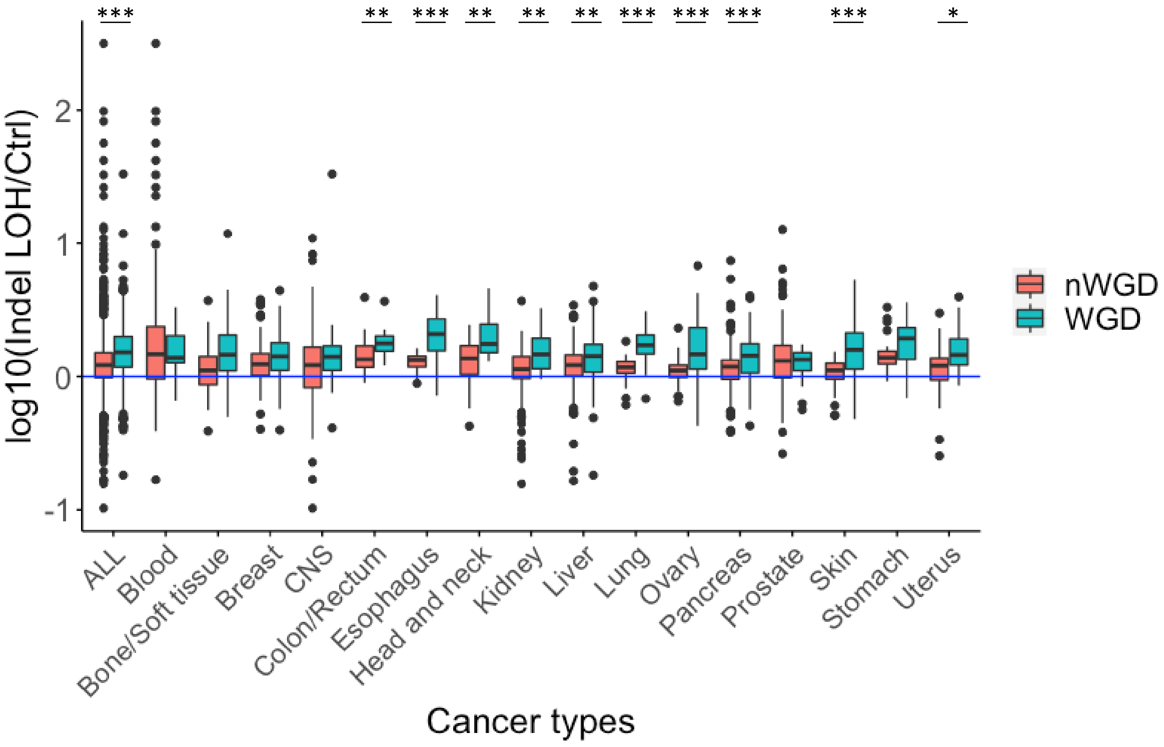

Supplementary Figure S2. The ratio of early clonal indel density within the LOH region to that outside the LOH region in WGD samples and the ratio of clonal indel density within the LOH region to that outside the LOH region in nWGD samples.

The vertical axis represents log10(indel density within the LOH region/indel density outside the LOH region). ALL indicates all samples without distinguishing cancer types.

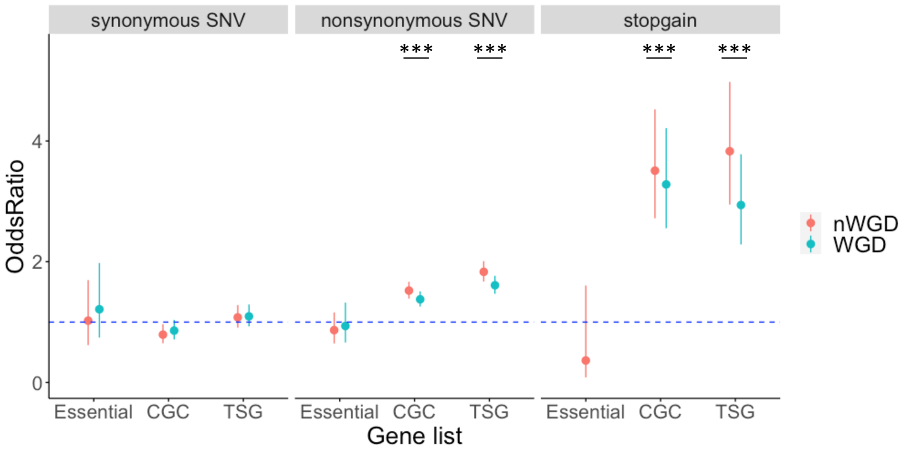

Supplementary Figure S3. Early mutation accumulation in genes within the LOH region.

The blue dotted line indicates an odds ratio of 1. Odds ratios of >1 indicate that the mutation is subjected to selective pressure. Red and blue dots represent odds ratios, whereas whiskers represent 95% confidence intervals.

Fig. S4
C Kikutake et. al

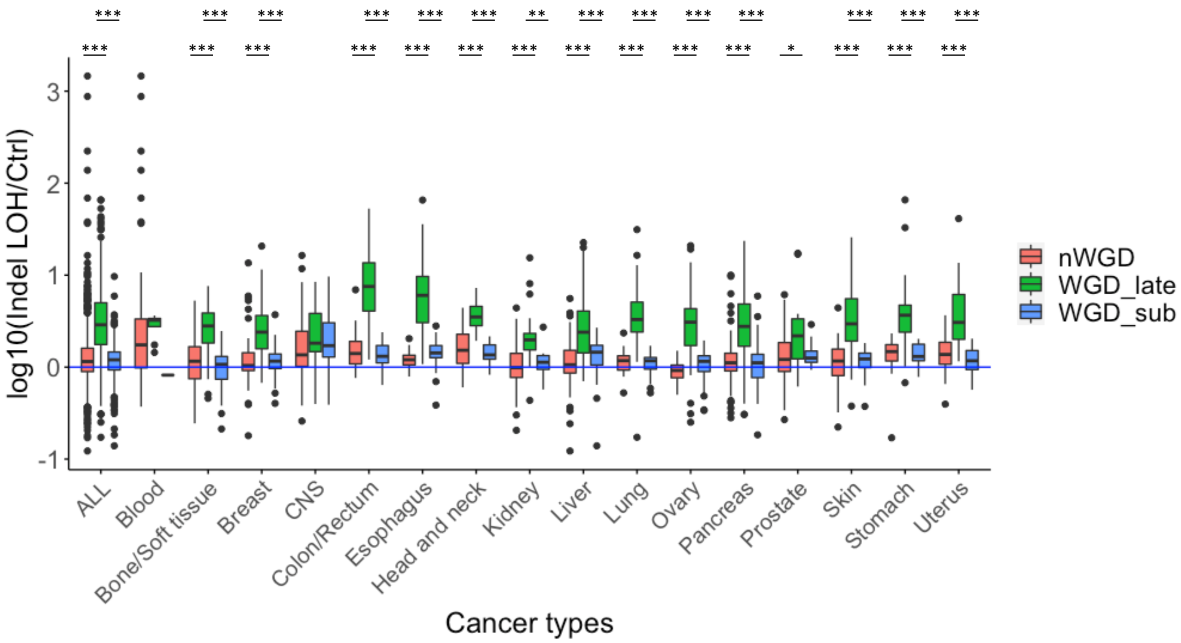

Supplementary Figure S4. The ratio of late clonal and subclonal indel density within the LOH region to that outside the LOH region in WGD samples and the ratio of subclonal indel density within the LOH region to that outside the LOH region in nWGD samples.

The vertical axis represents log10(indel density within the LOH region/indel density outside the LOH region). ALL indicates all samples without distinguishing cancer types.

Fig. S5
C Kikutake et. al

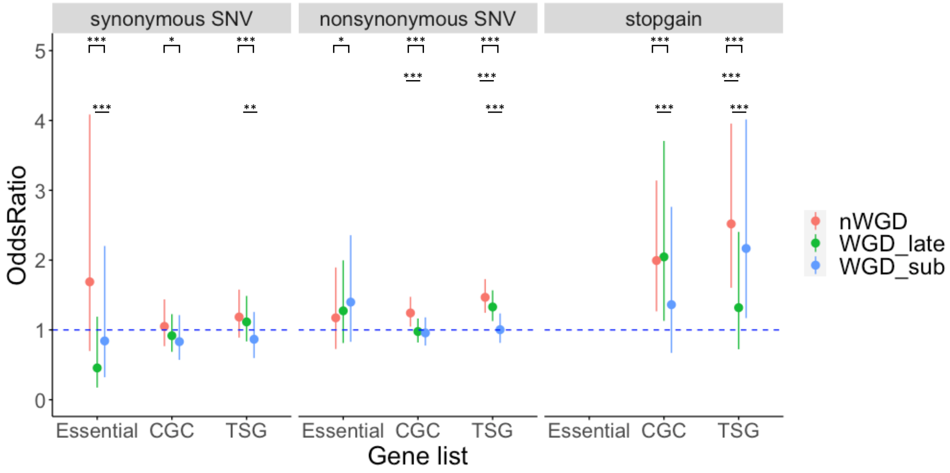

Supplementary Figure S5. Late mutation accumulation in genes within the LOH region.

The blue dotted line indicates an odds ratio of 1. Odds ratios of >1 indicate that the mutation is subjected to selective pressure. Red and blue dots represent odds ratios, whereas whiskers represent 95% confidence intervals.

Fig. S6
C Kikutake et. al

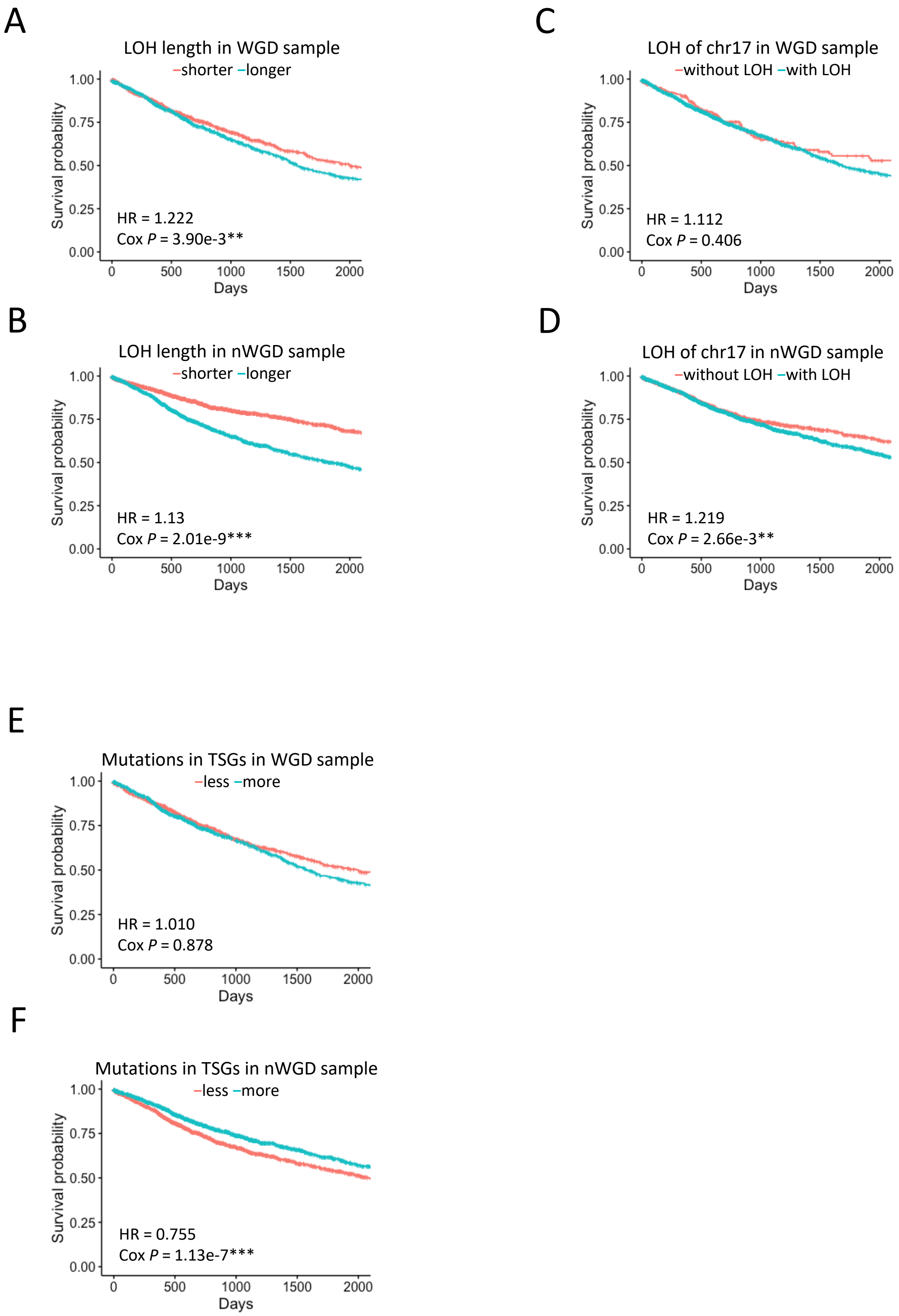

Supplementary Figure S6. Survival analysis using WGD occurrence-associated factors in case of early mutations based on TCGA data.

(A) Association between the length of LOH region and prognosis in WGD samples based on TCGA data. Samples were divided into two groups based on the median total length of LOH region in each sample: longer and shorter groups (blue and red lines, respectively). The horizontal and vertical axes represent survival days (days) and probability, respectively. (B) Association between length of LOH region and prognosis in nWGD samples based on TCGA data. The samples were divided into two groups based on the median total length of LOH region in each sample: longer and shorter groups (blue and red lines, respectively). (C) Association between LOH in chr17 and prognosis in WGD samples based on TCGA data. The samples were divided into two groups based on the presence or absence of LOH in chr17: groups with and without LOH (blue and red lines, respectively). (D) Association between LOH in chr17 and prognosis in nWGD samples based on TCGA data. The samples were divided into two groups based on the presence or absence of LOH in chr17: groups with and without LOH (blue and red lines, respectively). (E) Association between the number of early clonal mutations in TSGs in the LOH region and prognosis in WGD samples based on TCGA data. The samples were divided into two groups based on the median number of mutations in TSGs in the LOH region of each sample: groups with more and less mutations (blue and red lines, respectively). (F) Association between the number of clonal mutations in TSGs in the LOH region and prognosis in nWGD samples using TCGA data. The samples were divided into two groups based on the median number of mutations in TSGs in the LOH region of each sample: groups with more and less mutations (blue and red lines, respectively).

Fig. S7
C Kikutake et. al

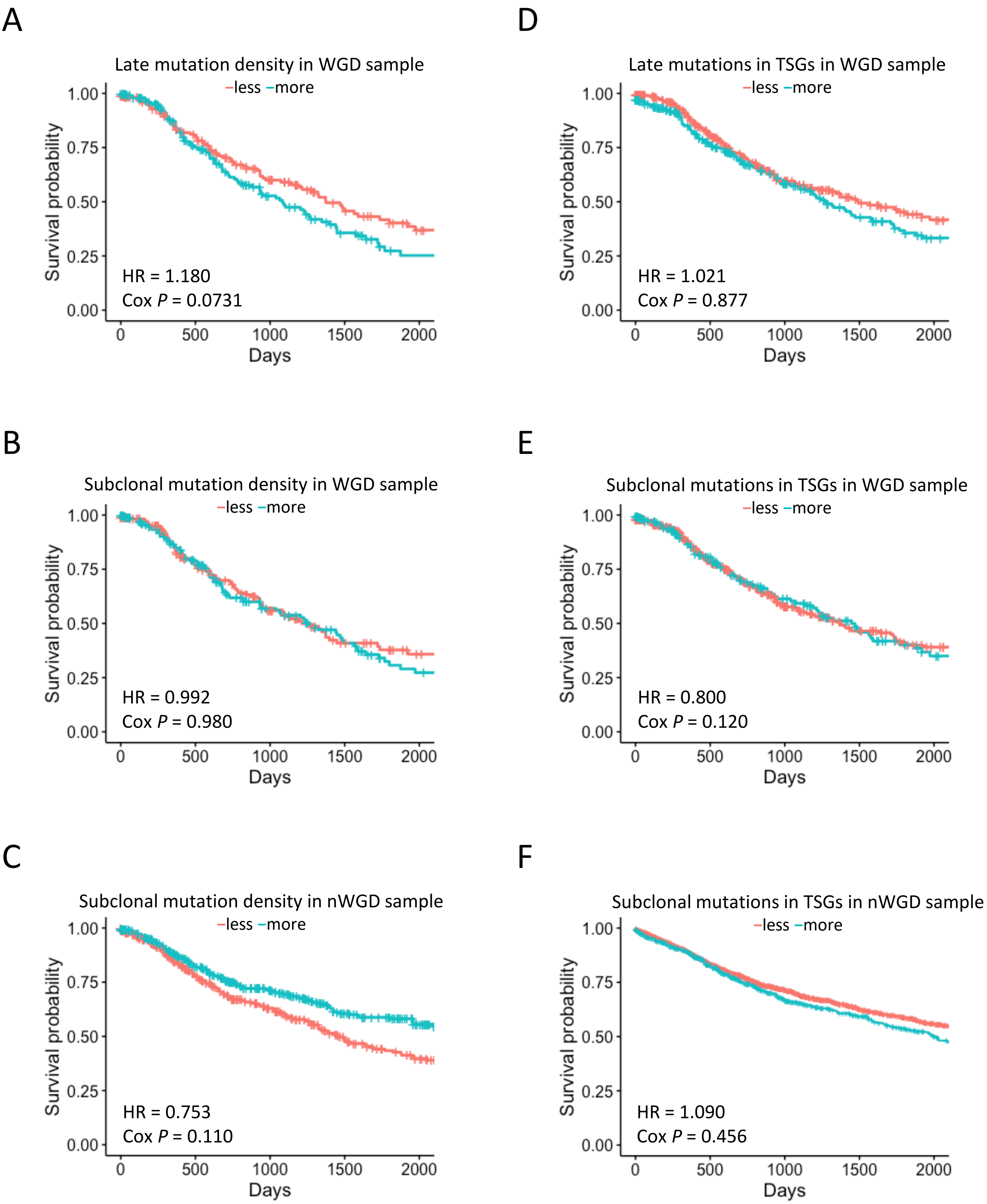

Supplementary Figure S7. Survival analysis using WGD occurrence-associated factors in case of late mutations.

(A) Association between the ratio of late clonal mutation density within the LOH region to that outside the LOH region and prognosis in WGD samples. The samples were divided into two groups based on the median ratio in each sample: higher and lower ratio groups (blue and red lines, respectively). The horizontal and vertical axes represent survival days (days) and probability, respectively. (B) Association between the ratio of subclonal mutation density within the LOH region to that outside the LOH region and prognosis in WGD samples. The samples were divided into two groups based on the median ratio in each sample: higher and lower ratio groups (blue and red lines, respectively). (C) Association between the ratio of subclonal mutation density within the LOH region to that outside the LOH region and prognosis in nWGD samples. The samples were divided into two groups based on the median ratio in each sample: higher and lower ratio groups (blue and red lines, respectively). (D) Association between the number of late clonal mutations in TSGs in the LOH region and prognosis in WGD samples. The samples were divided into two groups based on the median number of mutations in TSGs in the LOH region of each sample: groups with more and less mutations (blue and red lines, respectively). (E) Association between the number of subclonal mutations in TSGs in the LOH region and prognosis in WGD samples. The samples were divided into two groups based on the median number of mutations in TSGs in the LOH region of each sample: groups with more and less mutations (blue and red lines, respectively). (F) Association between the number of subclonal mutations in TSGs in the LOH region and prognosis in nWGD samples. The samples were divided into two groups based on the median number of mutations in TSGs in the LOH region of each sample: groups with more and less mutations (blue and red lines, respectively).

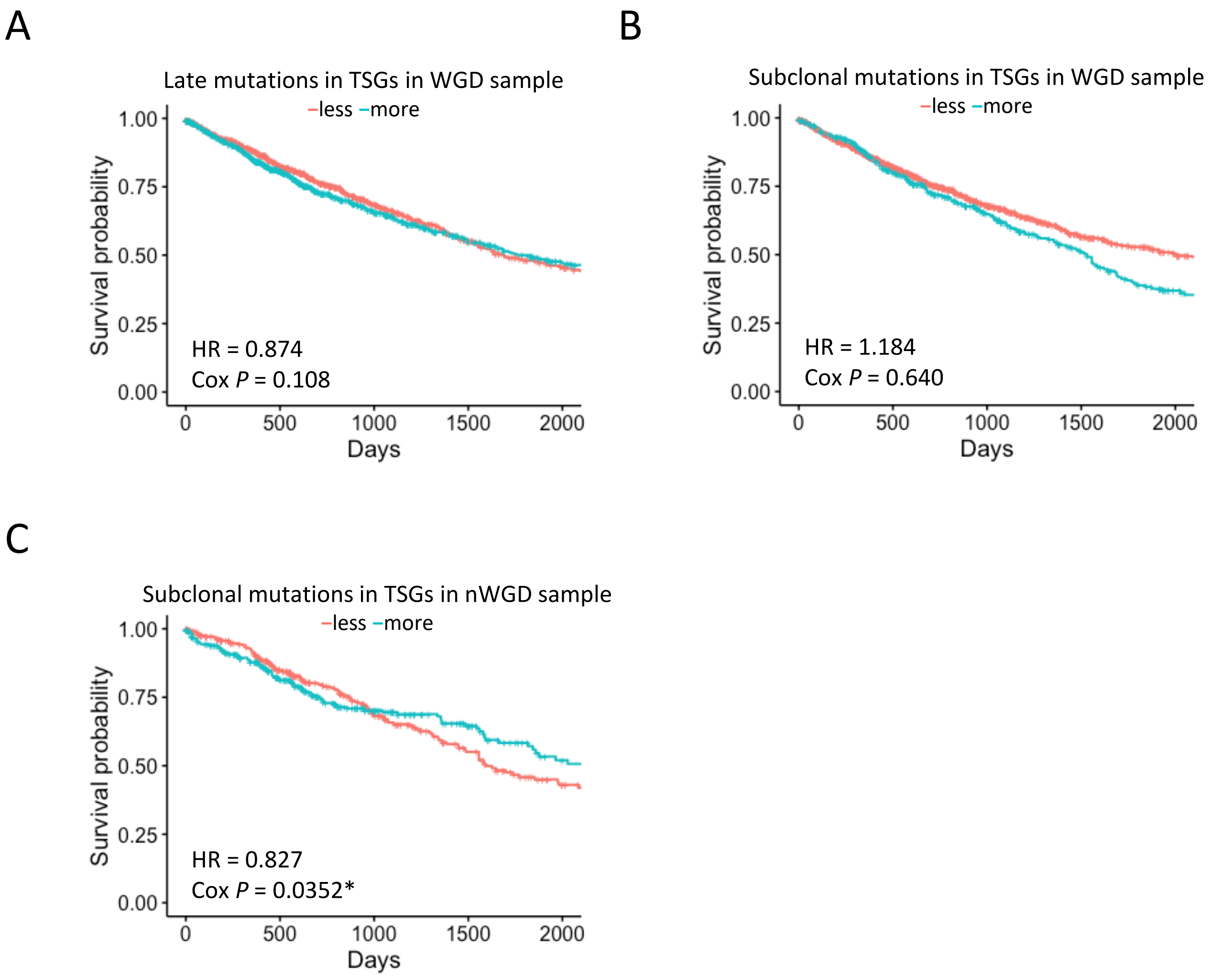

Supplementary Figure S8. Survival analysis using WGD occurrence-associated factors in case of late mutations based on TCGA data.

(A) Association between the number of late clonal mutations in TSGs in the LOH region and prognosis in WGD samples based on TCGA data. The samples were divided into two groups based on the median number of mutations in TSGs in the LOH region of each sample: groups with more and less mutations (blue and red lines, respectively). (B) Association between the number of subclonal mutations in TSGs in the LOH region and prognosis in WGD samples based on TCGA data. The samples were divided into two groups based on the median number of mutations in TSGs in the LOH region of each sample: groups with more and less mutations (blue and red lines, respectively). (C) Association between the number of subclonal mutations in TSGs in the LOH region and prognosis in nWGD samples based on TCGA data. The samples were divided into two groups based on the median number of mutations in TSGs in the LOH region of each sample: groups with more and less mutations (blue and red lines, respectively).

A

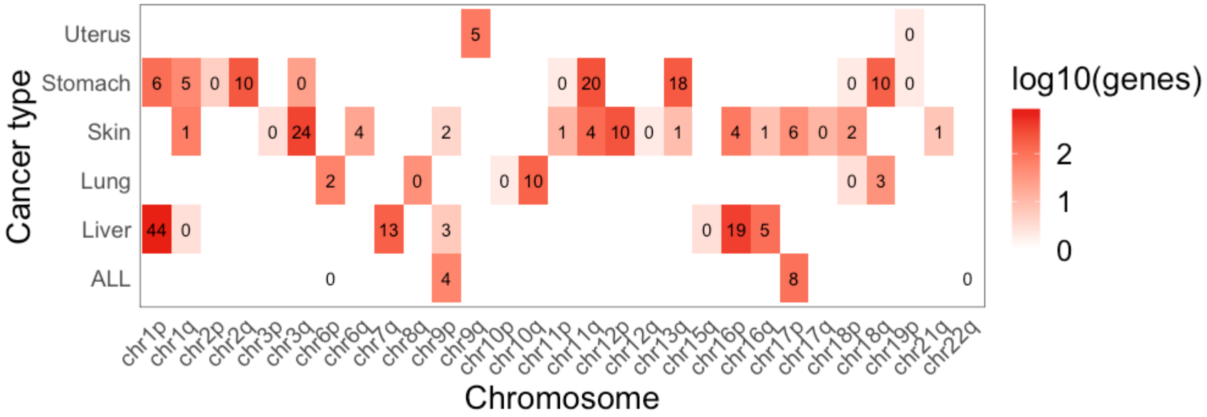

B

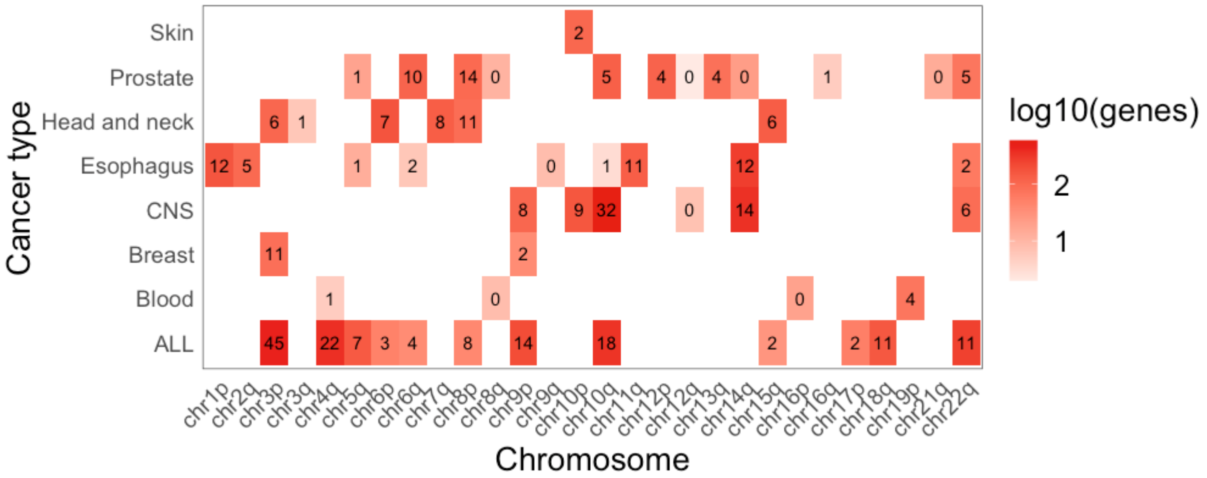

Supplementary Figure S9. Prognostic gene exploration using PCAWG data.

(A) The number of prognosis-related genes in the LOH region in WGD samples based on PCAWG data. These genes showed a worse prognosis when found in the LOH region than when not found in the LOH region. The red square indicates the number of genes. Numbers in the squares represent the number of TSGs. ALL indicates all samples without distinguishing cancer types. (B) The number of prognosis-related genes in the LOH region in nWGD samples using PCAWG data. ALL indicates all samples without distinguishing cancer types.

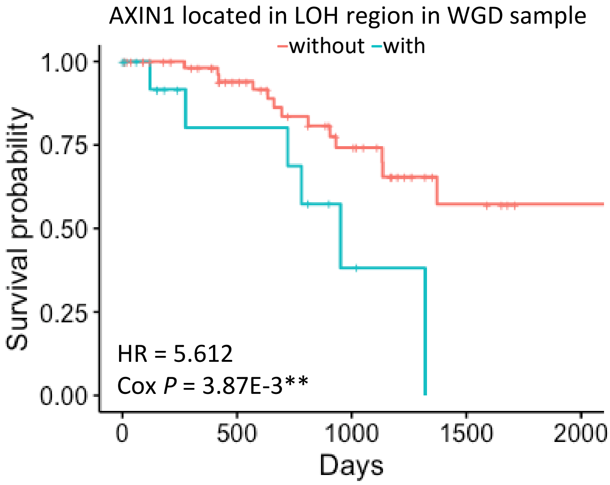

Supplementary Figure S10. Association between AXIN1 in the LOH region and prognosis in WGD samples using PCAWG data.

The samples were divided into two groups based on the presence or absence of AXIN1 in the LOH region: samples with AXIN1 in the LOH region (blue line) and samples without AXIN1 in the LOH region (red line). The horizontal and vertical axes represent survival days (days) and probability, respectively.

Fig. S11
C Kikutake et. al

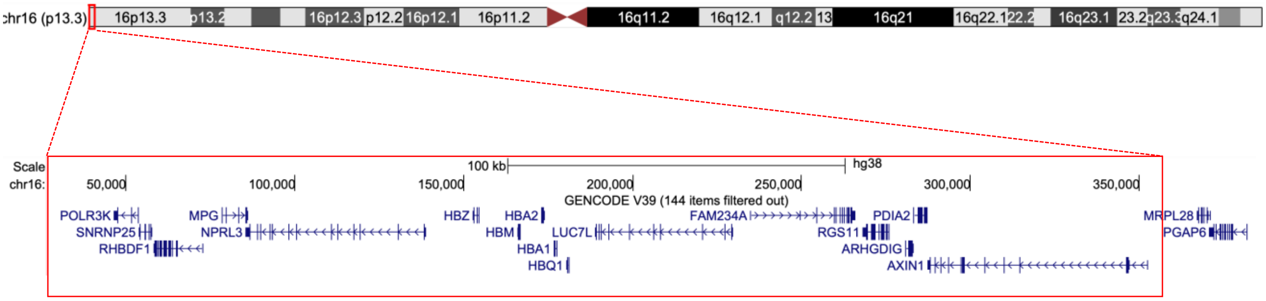

Supplementary Figure S11. Extracted prognosis-related genes in the 16p.13.3 locus.

The red square represents the 16 extracted prognosis-related genes in the 16p.13.3 locus.

Fig. S12
C Kikutake et. al

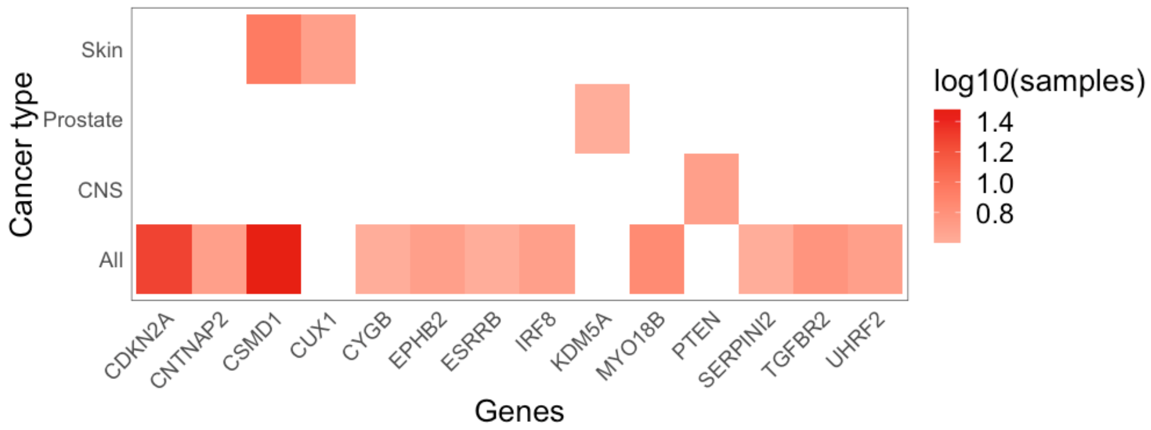

Supplementary Figure S12. Number of samples with prognosis-related TSGs in the LOH region of WGD samples using PCAWG data.

Samples with mutations in prognosis-related TSGs found in the LOH region represent a worse prognosis than other samples. The red square indicates the number of samples with mutations in genes found in the LOH region. ALL indicates all samples without distinguishing cancer types.

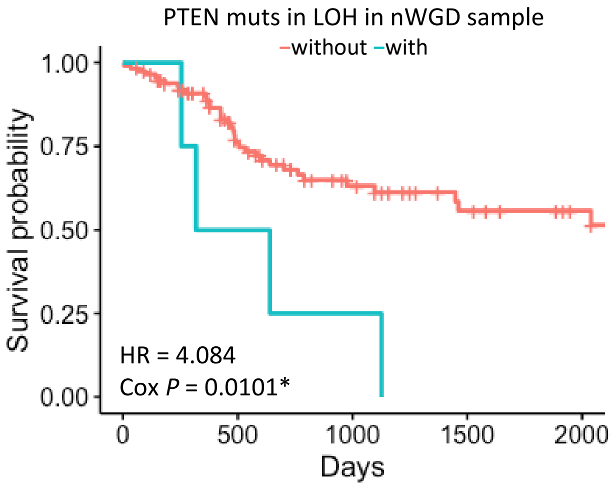

Supplementary Figure S13. Association between PTEN mutations in LOH and prognosis in nWGD samples using PCAWG data.

The samples were divided into two groups based on the presence or absence of PTEN mutations detected in the LOH region: samples with PTEN mutations (blue line) and samples without PTEN mutations (red line).
